# Ventral Hernia and Obesity: A Contemporary Surgical Challenge

**DOI:** 10.7759/cureus.83126

**Published:** 2025-04-28

**Authors:** Roberto Elías Damacio-Breton, Alfredo Sinahi Abarca-Magallon, Marco Aurelio Alvarez-Romero, Carlos Iskyam Zaldo-Arredondo, Jose Arturo Estrada-Gonzalez

**Affiliations:** 1 General Surgery, Hospital de Especialidades 5 de Mayo, Instituto de Seguridad y Servicios Sociales de los Trabajadores al Servicio de los Poderes del Estado de Puebla (ISSSTEP), Puebla, MEX; 2 Medicine, Benemerita Universidad Autonoma del Estado de Puebla, Puebla, MEX; 3 General and Colorectal Surgery, Hospital Regional Lic. Adolfo López Mateos, Instituto de Seguridad y Servicios Sociales de los Trabajadores del Estado (ISSSTE), Mexico City, MEX; 4 General Surgery, Hospital General de Leon, Leon, MEX; 5 General Surgery, Hospital General de Cancun, Cancun, MEX

**Keywords:** abdomen ventral hernia, bariatric surgery, incisional ventral hernia, obesity, retromuscular mesh repair

## Abstract

Ventral hernias are abdominal wall defects classified as primary or incisional. Obesity is a significant risk factor, contributing to wound healing impairment and abdominal wall weakness. We present a 37-year-old female patient with obesity (body mass index (BMI) 48.9) who developed incisional hernia (IH) one year after laparoscopic sleeve gastrectomy. Imaging revealed infraumbilical and paraumbilical IHs, leading to surgical repair with anterior component separation, unilateral transverse muscle release, and retromuscular mesh placement. Abdominoplasty was performed for functional and esthetic improvement. The patient had a favorable postoperative course. Managing IH in obese patients requires a multidisciplinary approach, considering specialized surgical techniques and long-term follow-up to minimize recurrence. This case highlights the interplay between bariatric surgery, hernia development, and the need for tailored preventive and therapeutic strategies.

## Introduction

Ventral abdominal hernias are defined as a non-inguinal, non-hiatal defect in the abdominal wall fascia [1]. In 2009, a consensus was reached by the European Hernia Society (EHS), where ventral hernias (VHs) were classified into primary VHs (PVHs) of the abdominal wall and incisional hernias (IHs) of the abdominal wall, defining the latter as any space in the abdominal wall with or without bulging in the area of a postoperative scar perceptible or palpable by clinical examination or imaging [2]. The incidence of IH after intra-abdominal surgery is approximately 15%-20% [3,4]. Risk factors related to the appearance of IH have been identified, which are related to previous disease (emergency surgery, midline incisions, and infection) [5,6], technical factors (closure technique, suture material), and patient-related factors (diabetes, obesity, smoking, and immunosuppression) [7], impacting wound healing alterations and abdominal wall weakness.

Obesity is a chronic disease characterized by excess adiposity that can induce alterations in organ and tissue function, increasing the risk of various pathologies and surgical complications [8]. Among these, VH is one of the most frequent complications in abdominal surgery, with a high postoperative recurrence rate [9]. Several studies have shown that obesity not only increases the incidence of VH but also represents a significant risk factor for its development. In particular, the distribution of adipose tissue, especially the increase in visceral adipose tissue and waist circumference, has been identified as a key determinant in the pathophysiology of this condition [9]. Furthermore, in the context of previous surgeries, such as in patients with colorectal cancer, it has been observed that a body mass index (BMI) greater than 30 significantly increases the risk of developing IHs, a subtype of VH, which underscores the relevance of considering obesity in surgical planning and the design of preventive strategies [4].

This report describes the surgical approach used to resolve a giant VH and reconstruct the abdominal wall in a patient with grade III obesity, previously subjected to laparoscopic sleeve gastrectomy. It details the anatomical and technical challenges derived from morbid obesity, as well as the surgical strategies used to optimize functional outcomes and reduce the risk of postoperative complications, highlighting the unique considerations in this patient population.

## Case presentation

A 37-year-old female patient with a significant medical history of three previous cesarean sections and an open cholecystectomy five years prior to evaluation presented for bariatric assessment due to an inability to lose weight, associated with lower back pain affecting her quality of life. She denied any history of diabetes mellitus or other significant comorbidities. A surgical procedure was considered a therapeutic alternative.

**Figure 1:**
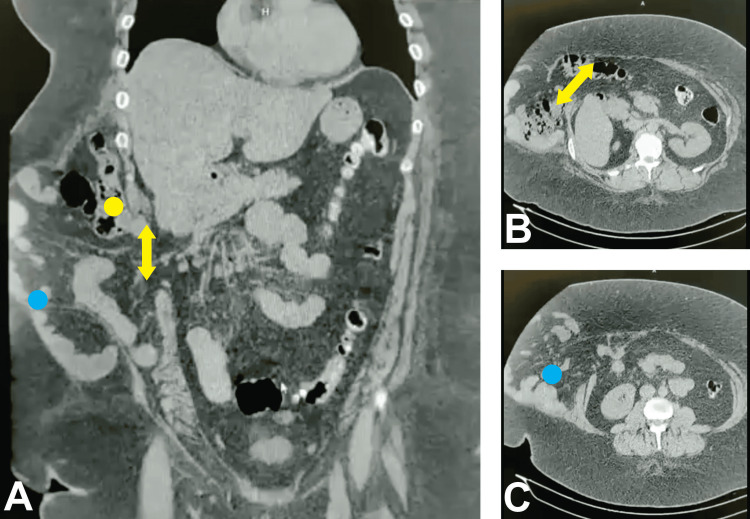
Preoperative Abdominal CT Imaging of the Incisional Hernia (A) Coronal plane demonstrating the hernial sac containing loops of the small intestine (blue dot) and colon (yellow dot) and the abdominal wall defect (yellow arrow, approximate width 12 cm). (B) Axial plane at the superior margin of the hernia defect (yellow arrow, approximate width 15 cm). (C) Axial plane at the inferior margin of the hernia defect (blue dot).

On physical examination, her anthropometric data showed a preoperative weight of 117.5 kg, a height of 155 cm, and a BMI of 48.9. Additionally, a right subcostal IH was identified. An abdominal CT scan confirmed a hernia defect measuring 15 x 12 cm, containing intestinal loops and the right colon (Figure 1).

A two-stage surgical approach was planned (Table 1). The first stage involved a laparoscopic sleeve gastrectomy, which was performed without complications.

**Table 1:** Two-Stage Surgical Approach BMI: body mass index

Stage	Procedure	Date	Key outcomes
1	Laparoscopic sleeve gastrectomy	May 2022	Significant weight loss achieved (BMI reduced from 48.9 to 29.6 kg/m^2^); no immediate complications
2	Abdominal wall reconstruction, abdominoplasty	April 2023	Successful hernia repair; improved abdominal contour; favorable postoperative course

**Figure 2:**
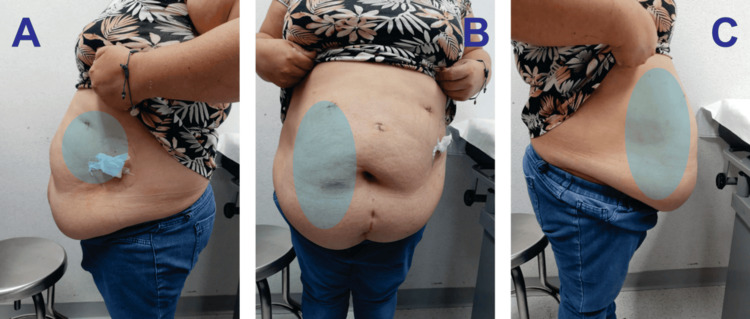
Preoperative Evaluation After Laparoscopic Sleeve Gastrectomy The images show postoperative changes from laparoscopic sleeve gastrectomy incisions and residual obesity with a right hemi-abdominal incisional hernia (highlighted in blue): (A) right lateral view, (B) anterior view, and (C) left lateral view.

**Figure 3:**
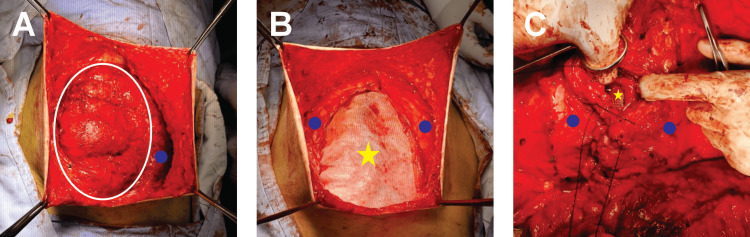
Abdominal Wall Reconstruction (A) Unilateral release of the transversus abdominis muscle indicated by blue dots and the herniary sac bordered by a white ring. (B) Retromuscular mesh placement (yellow star), retromuscular space. (C) Repaired abdominal wall.

Twelve months after the initial procedure, with improved anthropometric data (weight: 71 kg, BMI: 29.6) (Figure 2), the second stage was carried out. This included an abdominal wall reconstruction using the anterior component separation technique with unilateral release of the right transverse abdominal muscle and placement of a retromuscular mesh (Figure 3). Additionally, an abdominoplasty was performed with monobloc resection of skin and subcutaneous tissue, along with the placement of a subcutaneous Drenovac drain.

The patient reported that the hernia caused intermittent pain and discomfort, particularly with physical exertion. The decision to delay hernia repair until after significant weight loss was made to optimize surgical conditions and reduce the risk of recurrence.

**Figure 4:**
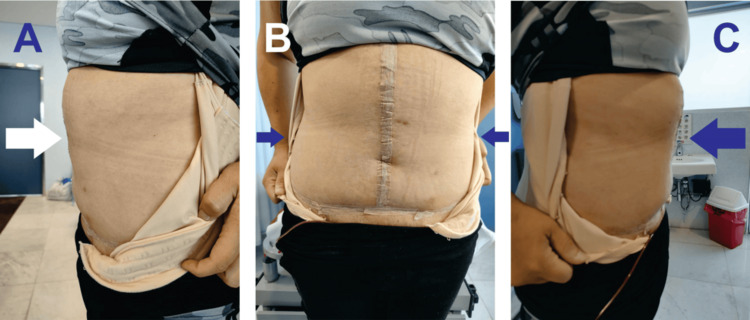
Early Postoperative Abdominal Wall Status (A) Right lateral view (white arrow indicates the garment's edge), (B) anterior view (blue arrows show the incision's extent), and (C) left lateral view (blue arrow indicates the garment's edge).

The postoperative course was favorable, with a hospital stay of three days and removal of the drain on the 10th postoperative day (Figure 4). The patient was mobilized on the first postoperative day and tolerated a regular diet by the second day. Pain was well-controlled with oral analgesics. There were no immediate surgical complications such as wound infection, seroma, or hematoma. At the six-month follow-up, the patient reported significant improvement in her quality of life, with resolution of her back pain and no evidence of hernia recurrence.

## Discussion

Obesity represents a significant risk factor for the development of VHs and IHs, as evidenced in the presented clinical case. The patient, with a preoperative BMI of 48.9 kg/m², had multiple risk factors that contributed to the development of IHs following bariatric surgery.

Obesity is defined as a condition characterized by excess adiposity, which may or may not be accompanied by abnormal adipose tissue distribution or function. It is considered a chronic and systemic disease that can alter tissue and organ function, leading to serious complications [8]. BMI, although useful for epidemiological studies and screening, can underestimate or overestimate adiposity, so it is recommended to confirm it with direct measurements of body fat or additional anthropometric criteria [8]. In patients with very high BMI (>40 kg/m²), as in the presented case, the presence of excess adiposity can be assumed [8].

Bariatric surgery, in this case, laparoscopic sleeve gastrectomy, was performed with the aim of achieving significant weight loss and improving the patient's quality of life. The success of this procedure was reflected in the reduction of BMI to 29.6 kg/m² at one year of follow-up. However, significant weight loss can also expose pre-existing weaknesses in the abdominal wall, which, added to the patient's surgical history, likely contributed to the development of IHs.

IHs are a type of VH that develops at the site of a previous surgical incision [2]. Obesity is a known risk factor for the development of VHs [9], and previous abdominal surgeries, such as open cholecystectomy, can significantly weaken the abdominal wall and predispose patients to IHs [4].

The management of IHs in patients with a history of obesity requires a multidisciplinary approach. The anterior component separation technique with unilateral release of the transversus abdominis muscle and retromuscular mesh placement, as performed in this case, are effective strategies to reinforce the abdominal wall and reduce the risk of recurrence [7]. Prophylactic mesh placement in high-risk patients undergoing elective laparotomy has also proven beneficial [3]. Although the patient was already undergoing laparoscopy during the bariatric procedure, concurrent intraperitoneal onlay mesh (IPOM) repair was not performed. This decision was based on concerns regarding the elevated risk of mesh infection in the setting of morbid obesity and the suboptimal conditions for achieving high-quality mesh fixation due to excessive intra-abdominal adiposity. Moreover, delaying the hernia repair allowed for significant weight loss, which optimized the abdominal wall tissue quality and reduced the risk of postoperative complications, ultimately enabling a more definitive and durable reconstruction. Finally, abdominoplasty with resection of redundant tissue contributed to improving both abdominal wall function and patient quality of life.

It is crucial to highlight the importance of long-term follow-up in patients with a history of obesity and abdominal surgery. Surveillance of the abdominal wall, weight optimization, and management of risk factors are essential to prevent the development of hernias and ensure successful surgical outcomes.

In summary, the concomitant management of obesity and VHs/IHs requires an individualized and multidisciplinary approach. Bariatric surgery can be an effective tool for achieving weight loss, but it is essential to consider the risk of hernia development and plan appropriate prevention and treatment strategies.

## Conclusions

This clinical case illustrates the complexity of managing VHs/IHs in patients with obesity. Obesity not only increases the risk of developing these hernias but also complicates their treatment. Bariatric surgery, while effective for weight loss, can predispose to the development of IHs, as observed in this case. Successful management requires a multidisciplinary approach, including specialized surgical techniques such as component separation and mesh placement, as well as long-term follow-up to prevent recurrences. The staged approach, with bariatric surgery followed by delayed hernia repair, can be a valuable strategy to optimize surgical conditions and improve outcomes in this challenging patient population. This approach highlights the importance of considering the impact of significant weight loss on abdominal wall integrity and the need for tailored surgical planning.

This case contributes to the existing literature by demonstrating the successful application of a combined surgical approach in a patient with a large IH and a history of morbid obesity and bariatric surgery. The findings suggest that this staged strategy is a feasible and effective option for managing such complex cases. For general and bariatric surgeons, this report emphasizes the need for careful preoperative assessment, consideration of the timing of hernia repair in relation to weight loss, and proficiency in advanced abdominal wall reconstruction techniques. While the specific techniques described may require specialized surgical expertise and resources, the principles of staged management and individualized treatment planning are applicable across various healthcare settings. However, the widespread adoption of this approach may be limited by factors such as access to bariatric surgery programs, availability of surgeons with expertise in complex hernia repair, and healthcare costs.
